# X-ray diffraction and TGA kinetic analyses for chemical looping combustion applications

**DOI:** 10.1016/j.dib.2017.12.044

**Published:** 2017-12-21

**Authors:** Mansour Mohammedramadan Tijani, Aqsha Aqsha, Nader Mahinpey

**Affiliations:** a Department of Chemical and Petroleum Engineering, Schulich School of Engineering, University of Calgary, Calgary, AB, Canada T2N 1N4; b Department of Chemical Engineering, Faculty of Engineering, Universiti Teknologi PETRONAS, 32610 Seri Iskandar, Perak Darul Ridzuan, Malaysia

## Abstract

Synthesis and characterization of supported metal-based oxygen carriers were carried out to provide information related to the use of oxygen carriers for chemical looping combustion processes. The Cu, Co, Fe, Ni metals supported with Al_2_O_3_, CeO_2_, TiO_2_, ZrO_2_ were prepared using the wetness impregnation technique. Then, the X-ray Diffraction (XRD) characterization of oxidized and reduced samples was obtained and presented. The kinetic analysis using Thermogravimetric analyzer (TGA) of the synthesized samples was conducted. The kinetics of reduction reaction of all samples were estimated and explained.

**Specifications Table**

**Table:** 

Subject area	Chemical Engineering, Energy, Environment, Material Science, Catalysis.
More specific subject area	Chemical Looping Combustion (CLC), Carbon Capture, Metal-based oxygen carriers.
Type of data	Images (x-ray, TGA kinetic calculations).
How data was acquired	X-ray Diffraction: Rigaku ULTIMA III X-ray diffractometer, TGA: TG 209 F1 Libra.
Data format	Analyzed.
Experimental factors	Dried at 120 °C for 12 h, calcined in air at 500 °C for 3 h, reduced with hydrogen gas (50 ml/min) at 350 °C for 3 h
Experimental features	Oxidized and reduced samples were obtained, then analyzed with the XRD. The CLC-TGA reactivity assessment was carried out (800 °C–950 °C).
Data source location	University of Calgary, Calgary, Alberta, T2N 1N4, Canada.
Data accessibility	Data are presented in this article.
Related research article	Mansour Mohammedramadan Tijani, Aqsha Aqsha, and Nader Mahinpey. Synthesis and study of metal-based oxygen carriers (Cu, Co, Fe, Ni) and their interaction with supported metal oxides (Al_2_O_3_, CeO_2_, TiO_2_, ZrO_2_) in a chemical looping combustion system. Energy. 2017; 138(C): 873–882.

**Value of the data**

• The data represent characterization of catalysts in term of different metal phases that existed during calcination and reduction experiments of metal-based oxygen carriers for CLC applications.
• The data show essential calculations used to estimate the kinetics of metal-based oxygen carriers for methane fueled CLC process.
• The data are useful for further studies on the development of kinetic models and determining the mechanism of reactions in the CLC process.

## 1 Data

The data present the XRD analysis of metal-based oxygen carriers for CLC applications. The data are Supplementary materials for the study describing the “Synthesis and study of metal-based oxygen carriers (Cu, Co, Fe, Ni) and their interaction with supported metal oxides (Al_2_O_3_, CeO_2_, TiO_2_, ZrO_2_) in a chemical looping combustion system” [1].

**Fig. 1:**
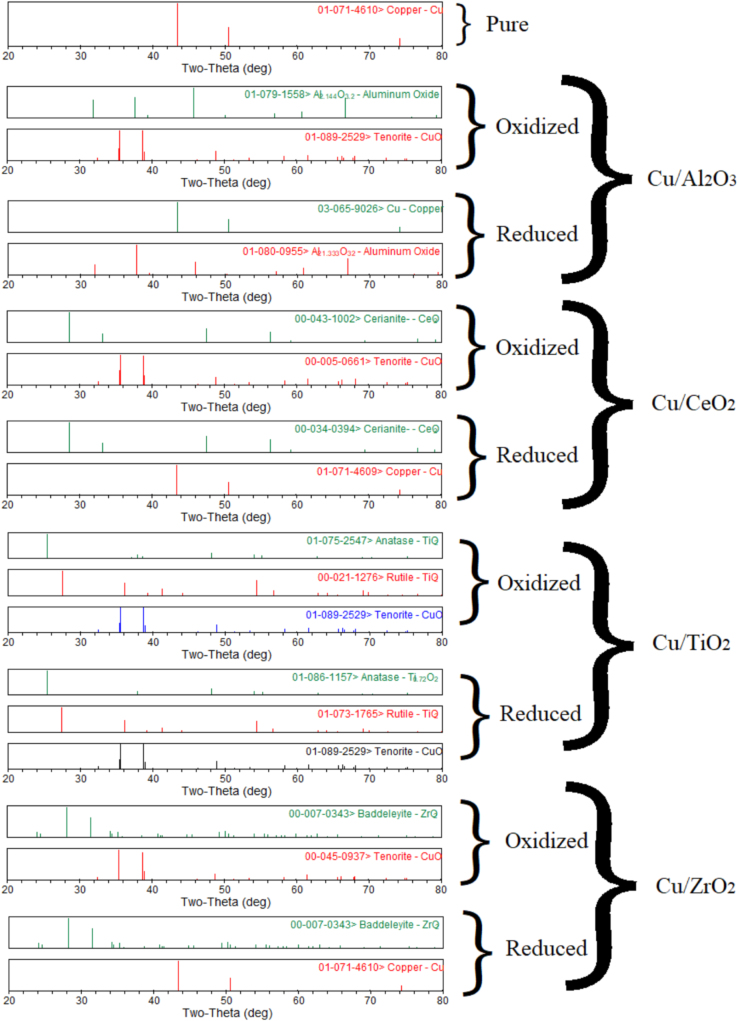
Metal phases existed in Cu-based oxygen carriers using XRD analysis.

**Fig. 2:**
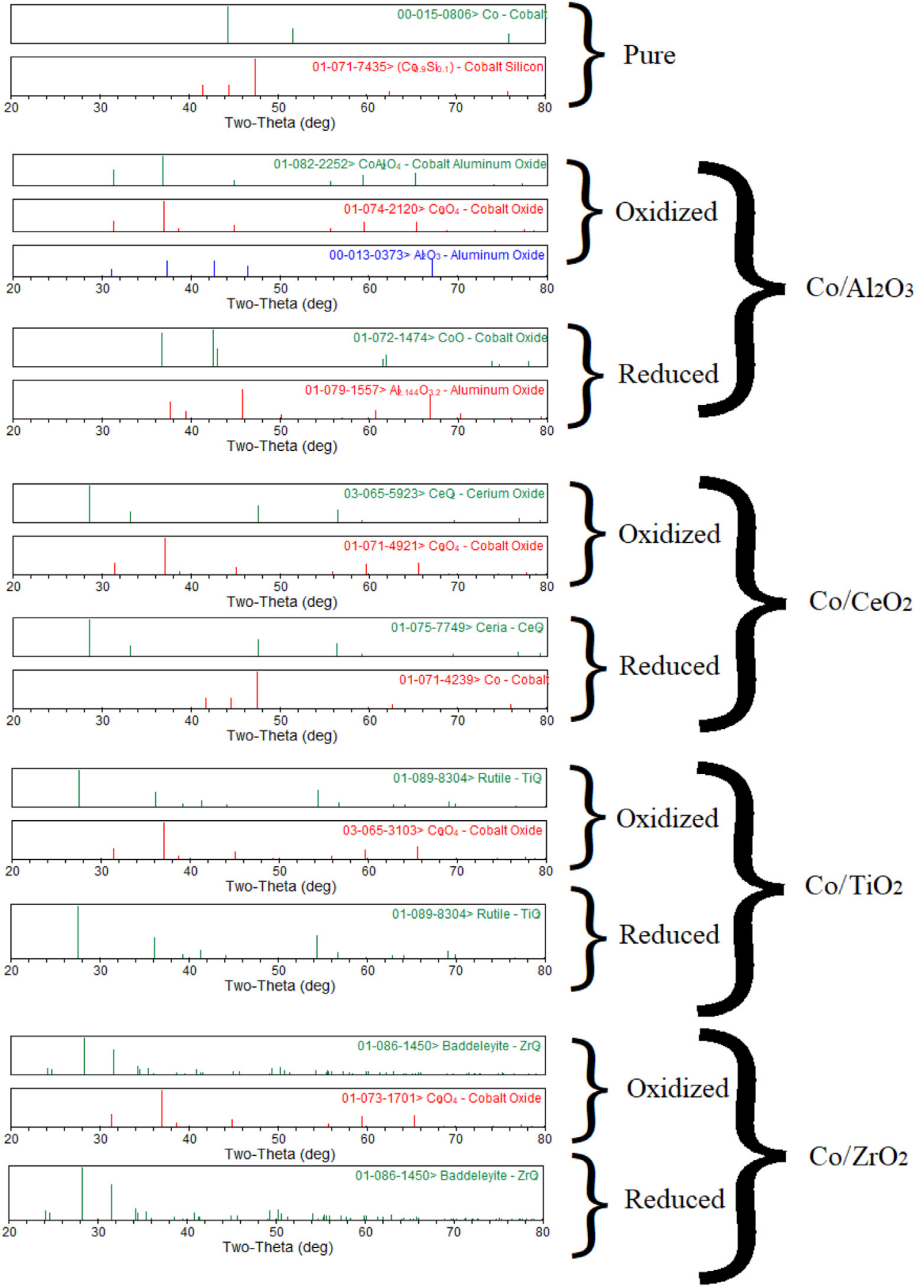
Metal phases existed in Co-based oxygen carriers using XRD analysis.

**Fig. 3:**
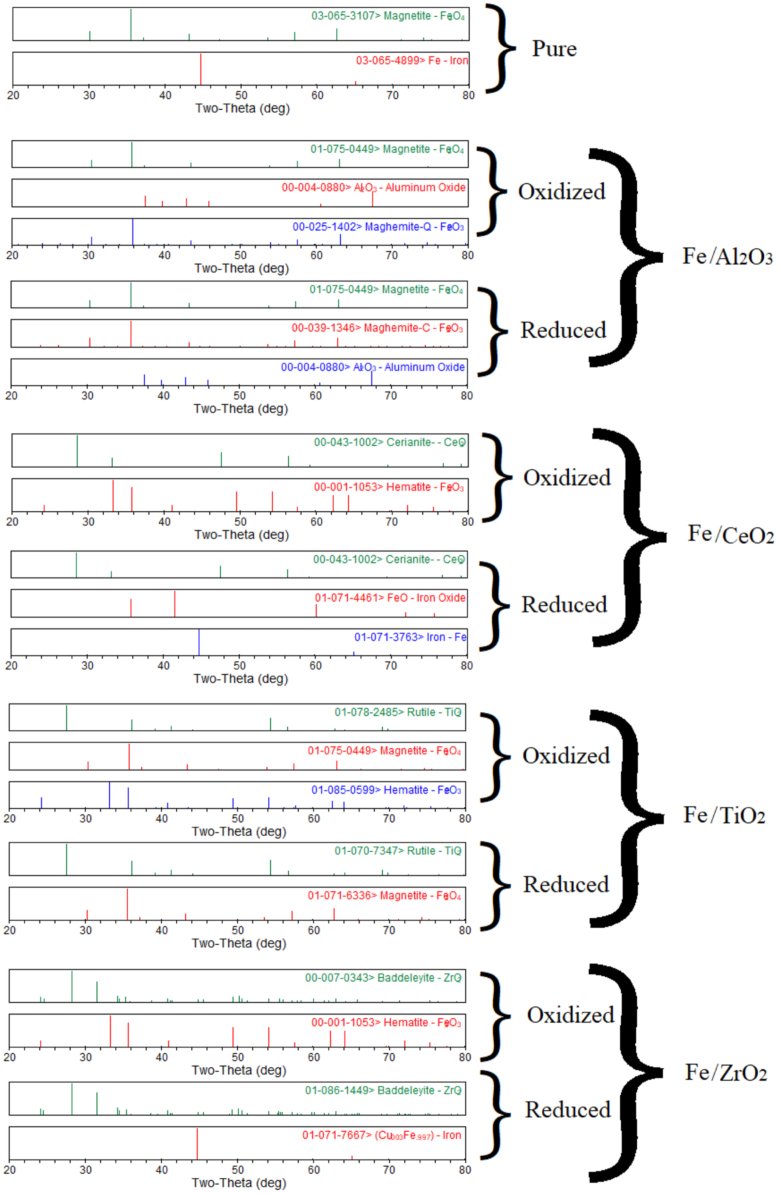
Metal phases existed in Fe-based oxygen carriers using XRD analysis.

**Fig. 4:**
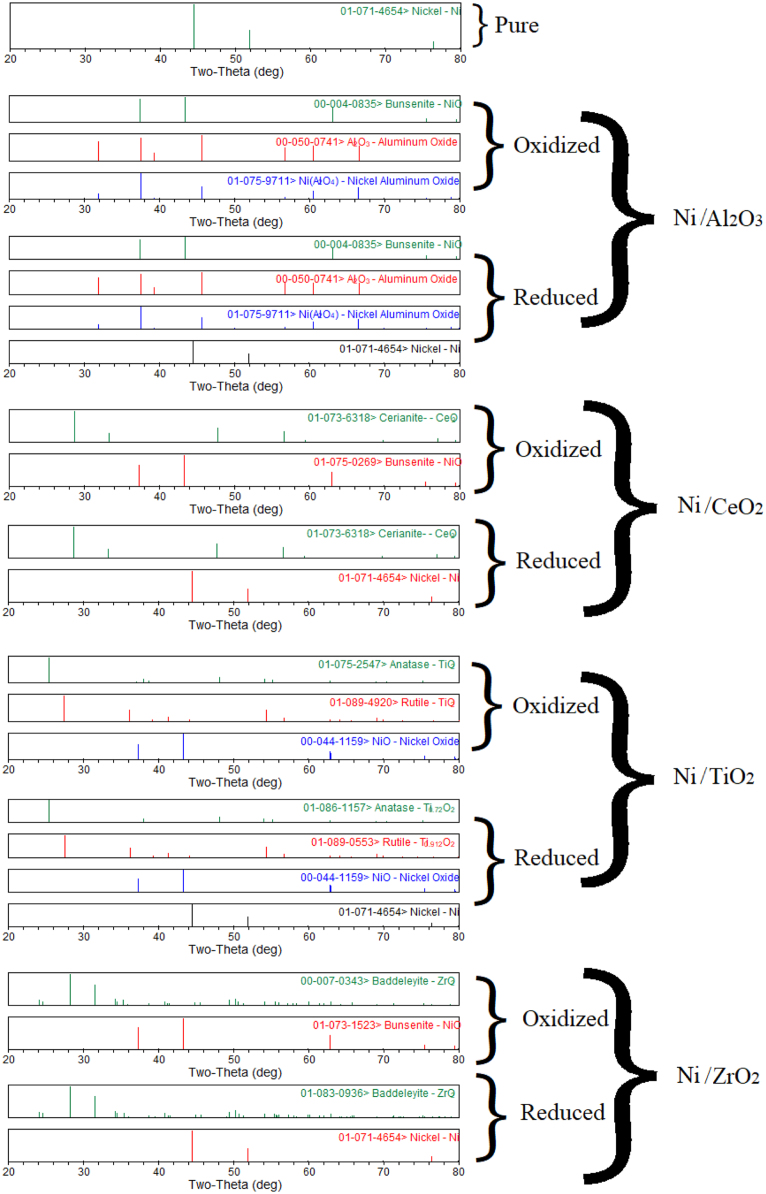
Metal phases existed in Ni-based oxygen carriers using XRD analysis.

The XRD analysis of Co, Cu, Fe, Ni metals supported with Al_2_O_3_, CeO_2_, TiO_2_, ZrO_2_ is shown in Fig. 1, Fig. 2, Fig. 3, Fig. 4. It was reported a complete reduction of CuO supported with Al_2_O_3_, CeO_2_, and ZrO_2_ (Fig. 1). CuO supported with TiO_2_ did not reduce to Cu under this experimental condition. The phases existed in the oxidized sample of Co supported with Al_2_O_3_ (Fig. 2) were Co_3_O_4_, Al_2_O_3_, and CoAl_2_O_4_, while only phases existed in the reduced sample were CoO, and Al_2_O_3_ phases. New phase of CoAl_2_O_4_ was observed in the oxidized sample of Co supported with Al_2_O_3_. A complete reduction of Co_3_O_4_ supported with CeO_2_ was observed. No trace of Co was noticed in Co sample supported with TiO_2_ and ZrO_2_. The Fe sample supported with Al_2_O_3_ (Fig. 3) did not reduce under this experimental condition. A partial reduction of Fe_2_O_3_ sample supported with CeO_2_ and TiO_2_ was noticed, while a complete reduction of Fe_2_O_3_ to Fe occurred in the Fe sample supported with ZrO_2_. No significant phase changes between the oxidized and reduced samples were observed in the NiO/Al_2_O_3_ (Fig. 4) sample; however, an intermediate phase (NiAl_2_O_4_) existed in both oxidized and reduced samples. A complete reduction of NiO supported with CeO_2_ and ZrO_2_ was observed. A partial reduction of NiO to Ni was found in the Ni sample supported with TiO_2_.

The weight loss and gain during the CLC reaction in the TGA were recorded and analyzed to study the effect of temperature on the conversion of Cu, Co, Fe, and Ni samples. The conversion of the reduction reaction of all samples was calculated using the following equation:

(1) $$X=\frac{m-m_{r}}{m_{o}-m_{r}}$$

where; m is the mass of sample at any time (g), $m_{r}$ is mass of the reduced sample (g); and, $m_{o}$ is the mass of the oxidized sample (g).

The conversion profiles during the reduction reaction showed no specific trend for all samples. However, a kinetic model that was developed by Gomez and Mahinpey [2] could be used to estimate the kinetic parameters of the reduction reaction. Considering that the surface reaction was the controlling step; hence, the equation used [2]:

(2) $$1/t_{X}\left(T\right)=k_{o}-G(X)+e^{\frac{{-E}_{r}}{\mathrm{RT}}}$$

where; t_X_ is residence time (min); X is conversion (-); T is absolute temperature (K); G(X) is conversion dependent function (-); k_o_ is the frequency factor (1/min); E_r_ is activation energy of reduction reaction (J/mol); and, R is the universal gas constant (J/mol K).

If the reaction rate at a constant conversion is only a function of temperature, the following assumption applies [2]:

(3) $$\ln\left[G(X)\right]\ll \ln\left[k_{o}\right]$$

**Fig. 5:**
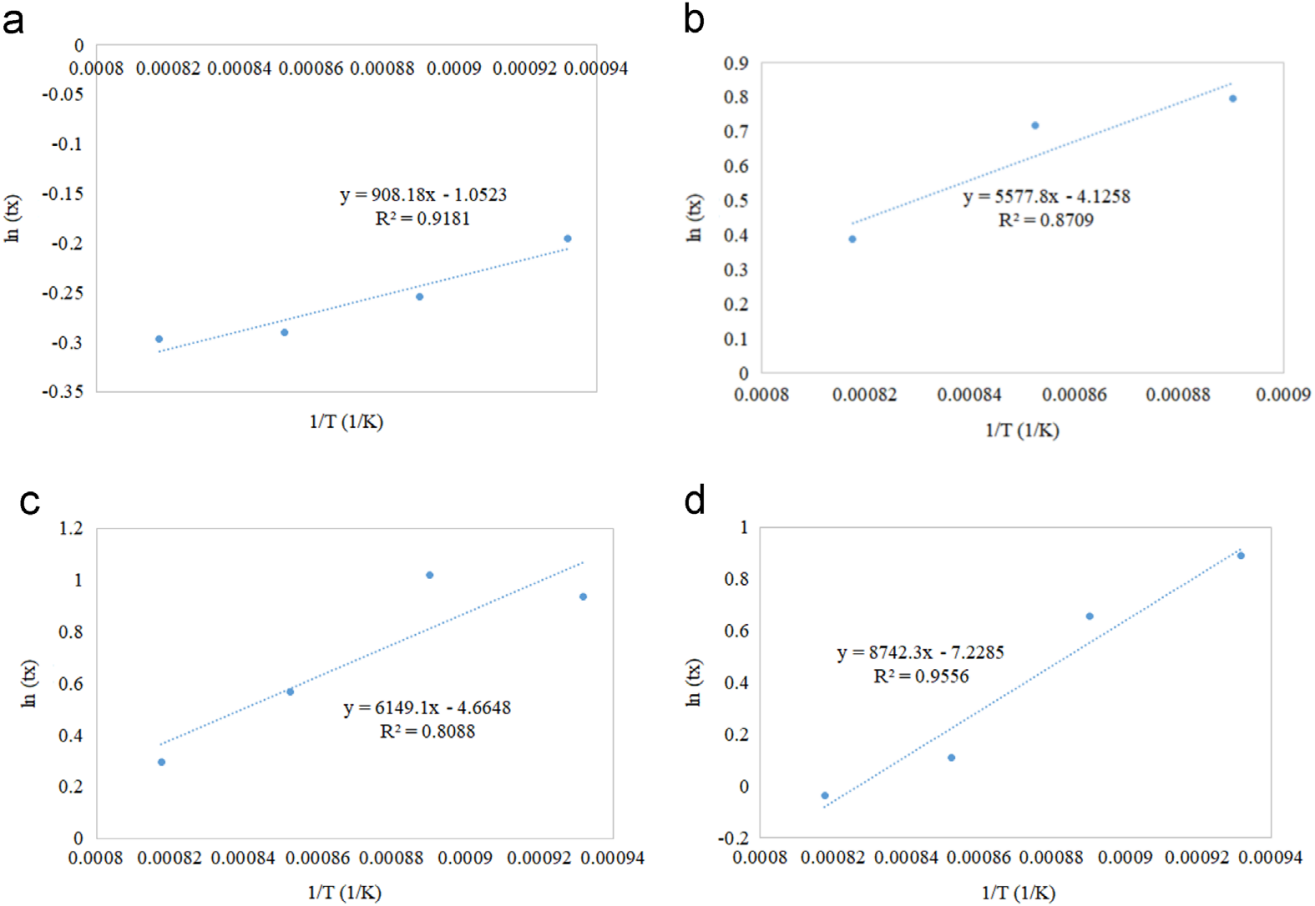
Residence time as a function of reciprocal of reaction temperature for (a) Cu/Al_2_O_3_ (b) Cu/CeO_2_ (c) Cu/TiO_2_ (d) Cu/ZrO_2_.

**Fig. 6:**
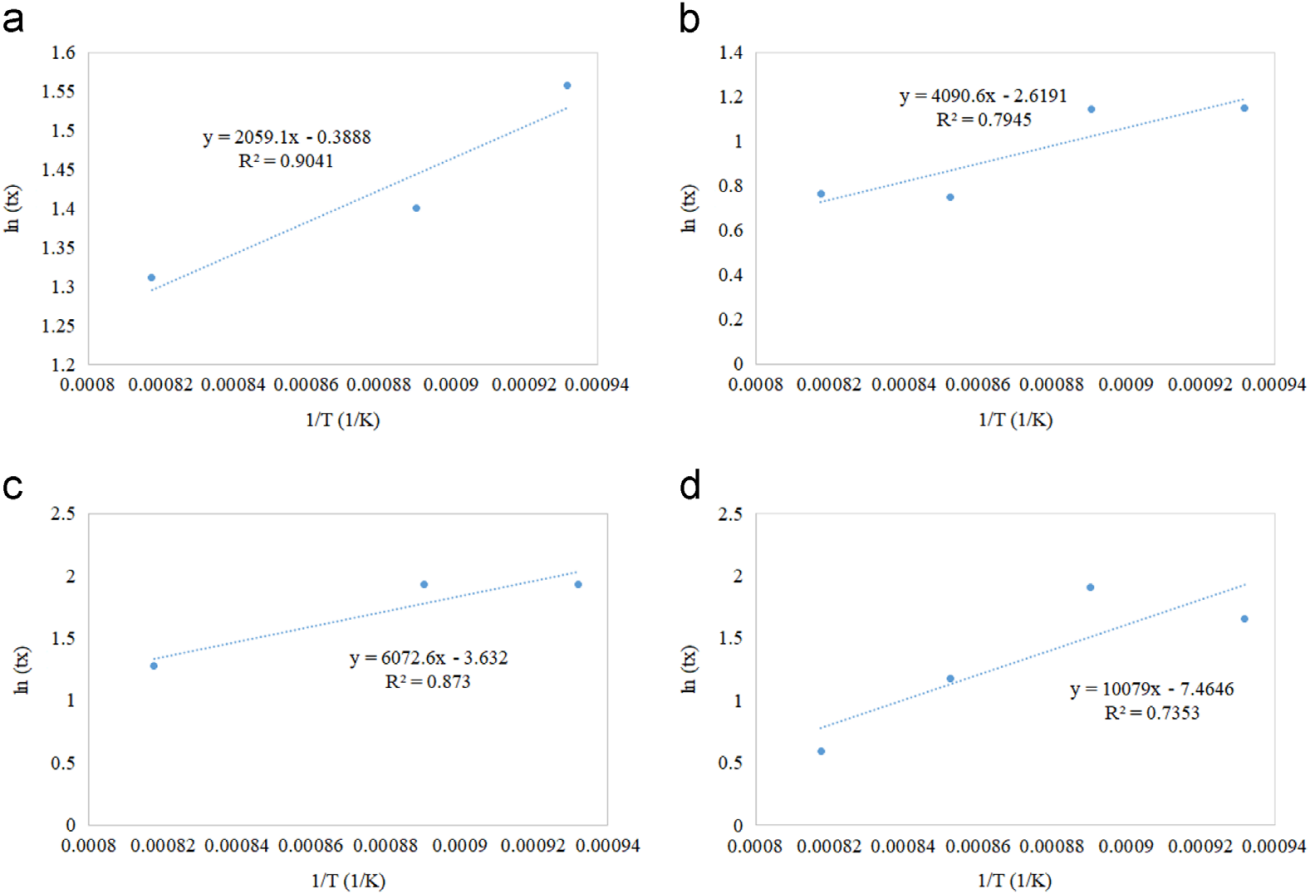
Residence time as a function of reciprocal of reaction temperature for (a) Co/Al_2_O_3_ (b) Co/CeO_2_ (c) Co/TiO_2_ (d) Co/ZrO_2_.

**Fig. 7:**
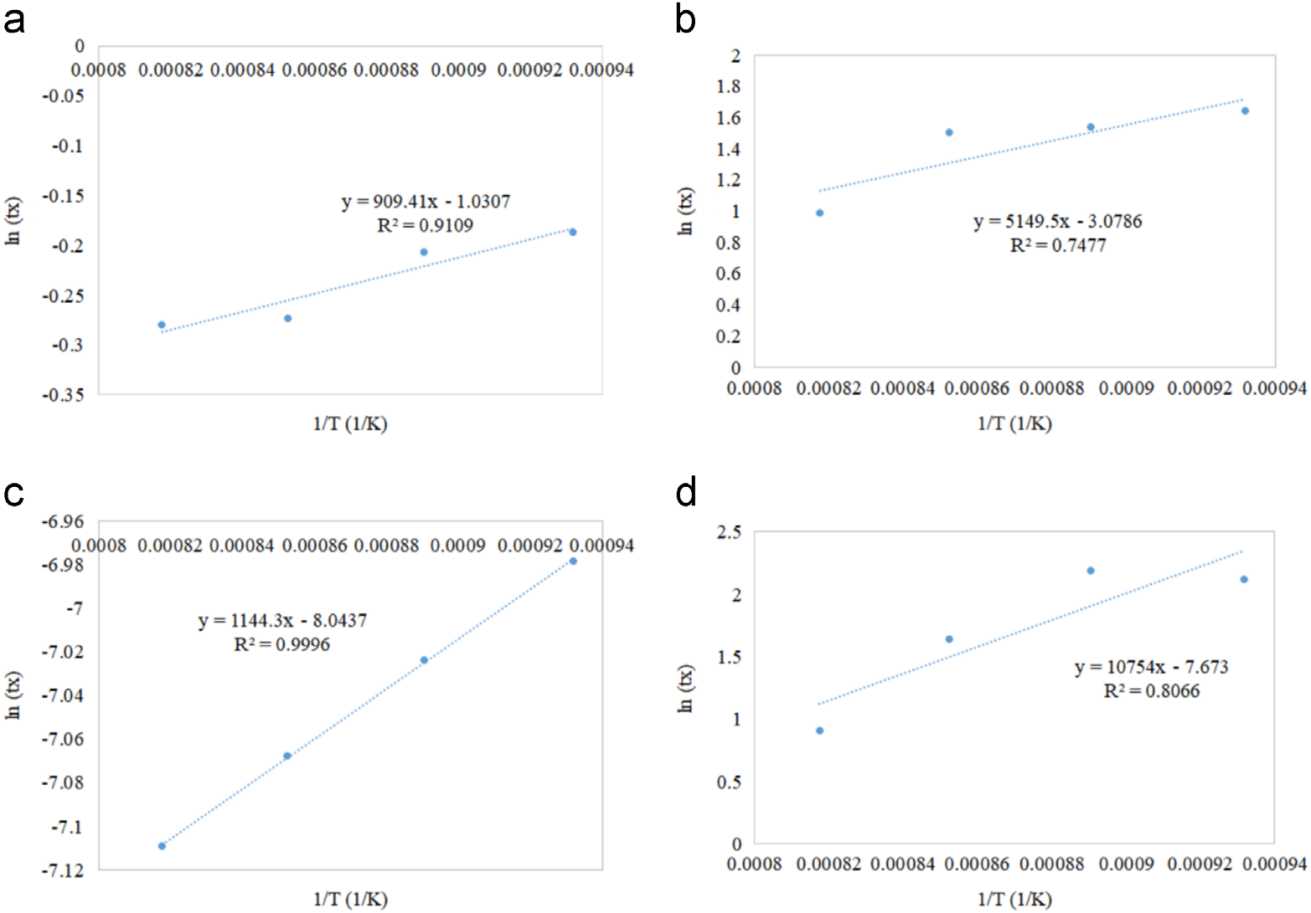
Residence time as a function of reciprocal of reaction temperature for (a) Fe/Al_2_O_3_ (b) Fe/CeO_2_ (c) Fe/TiO_2_ (d) Fe/ZrO_2_.

**Fig. 8:**
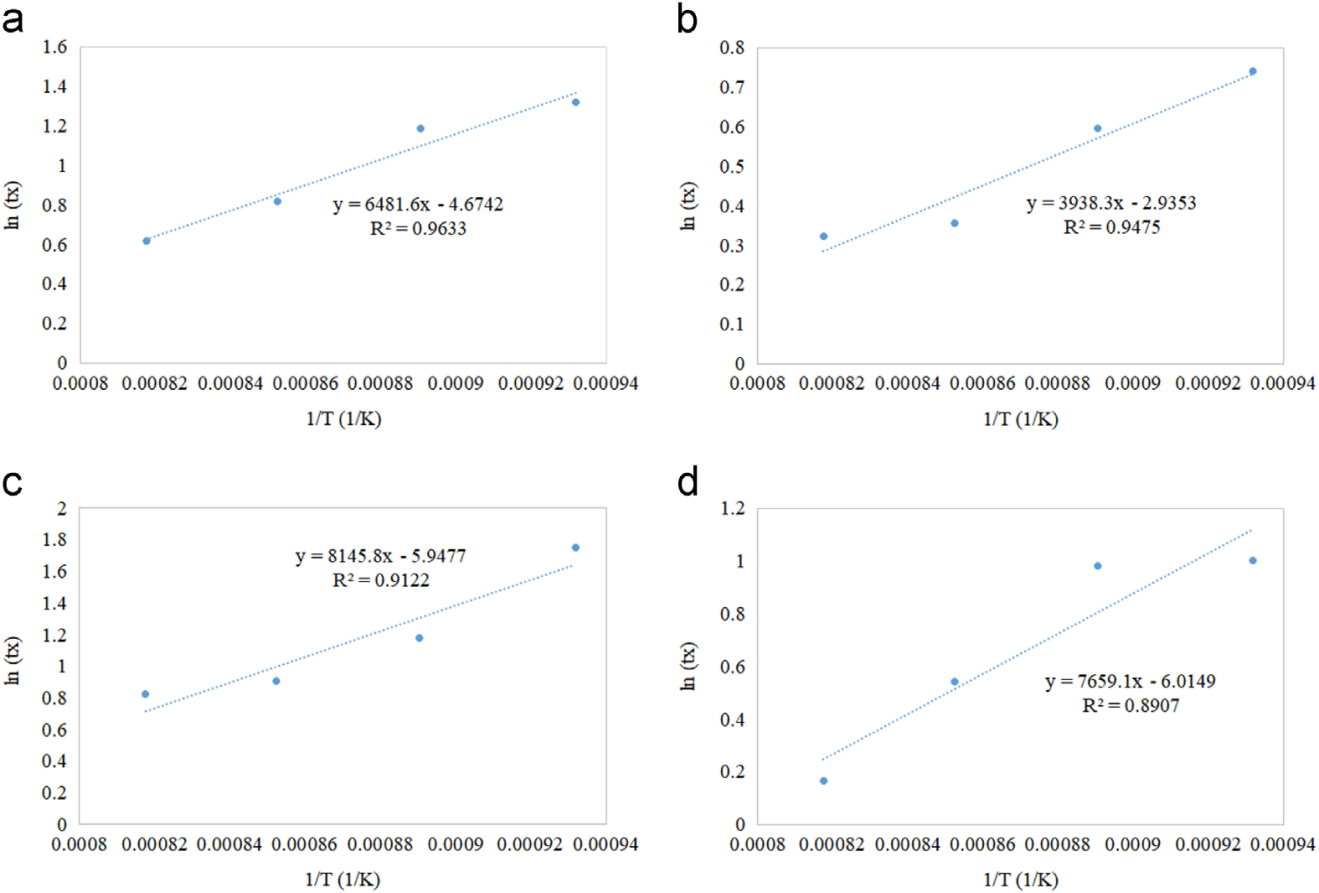
Residence time as a function of reciprocal of reaction temperature for (a) Ni/Al_2_O_3_ (b) Ni/CeO_2_ (c) Ni/TiO_2_ (d) Ni/ZrO_2_.

The following graphs (Fig. 5, Fig. 6, Fig. 7, Fig. 8) were generated for each sample to estimate Er and k_o_:

All supported oxygen carriers exhibited an increase in the residence time as temperature decreased to achieve 50% solid conversion. Fast reduction profiles compared to other supported samples were noticed in the reduction reaction of all Cu-based oxygen carriers. As the temperature increased, the reaction rate increased but reduction time decreased. Similar reduction behaviour of Cu-based oxygen carriers was observed in the supported Co-based (Fig. 6), Fe-based (Fig. 7), and Ni-based (Fig. 8) oxygen carriers. However, the reduction of Fe (Fig. 7) exhibited an additional resistant step that could be due to the deep reduction (i.e. phase transitions from FeO to Fe, Fe_2_O_3_ to FeO and/or Fe_2_O_3_ to Fe_3_O_4_). The Cu/Al_2_O_3_ sample showed a complete reduction time between 1 to 1.5 min during the first cycle of CLC, while other supported Cu samples with CeO_2_, TiO_2,_ and ZrO_2_ showed a complete reduction time of more than 5 min The Co/Al_2_O_3_ sample showed a complete reduction time of less than 5 min during the first cycle of CLC. The other supported Co samples by CeO_2_, TiO_2_, and ZrO_2_ showed a fluctuated reduction time that ranged from 2.5 to 10 min The Fe/ZrO_2_, and Fe/Al_2_O_3_ samples showed the fastest reduction rates (90% in only 1.5 min) compared to other Fe supported oxygen carriers. The Ni/ZrO_2_ sample reported a complete reduction time of 3 min.

Referring to Eq. (2), the slope in Fig. 5, Fig. 6, Fig. 7, Fig. 8 represents the $E_{r}/R$ term in which the activation energy of the reduction reaction ($E_{r}$) was estimated by multiplying the slope with the universal gas constant. The intercept in Fig. 5, Fig. 6, Fig. 7, Fig. 8 represents $\ln\left[k_{o}\right]$ term in which the frequency factor was estimated by taking the logarithmic inverse.

## 2 Experimental design, materials, and methods

The metal-based oxygen carriers were prepared using the incipient wetness impregnation method [3] at atmospheric pressure.

The following units were obtained beforehand and thoroughly cleaned:

• Digital scale
• Glass beaker (size: 250 ml)
• Magnetic stirrer
• Spatula
• Metal nitrates
• Support oxides
• Hotplate/stirrer
• DI water
• Ceramic mortar/bowl

The molar calculations performed based on the required percentage of both active metals and supports to determine the exact required mass. Then, in a beaker/magnetic stirrer, a 50 ml of DI water was added and the magnetic stirrer launched to a speed of 400-rpm. The appropriate amount of the metal nitrates obtained in a weighting paper and then carefully added to the beaker. The sample left to dissolve for 10 min. Next, a 100 ml of DI water was added to the beaker and the appropriate amount of the support oxides was weighted and carefully added to the beaker. The beaker was then covered and left stirred for 24 h at room temperature. Next, heating of the beaker started until the temperature of solution reached to 75 °C. Most of water evaporated and the muddy sample was then collected into a ceramic bowl using a spatula. The sample dried in a conventional oven for 12 h at 120 °C. Crushing of the dried samples using a ceramic mortar/bowl into a fine powder-like was accomplished. Finally, the dried sample calcined in air at 500 °C for 3 h and reduced with hydrogen gas (50 ml/min) through a stainless-steel tubular reactor at 350 °C for 3 h.

The X-ray diffraction (XRD) analysis was conducted using a Rigaku ULTIMA III X-ray diffractometer with Cu K-alpha radiation. The oxygen carriers scanned with 2-theta equal to 20–80, a 0.05° step, and a counting time of 2.0° per min, operating at 40 kV and 44 mA.
